# Supplementary material for: The acceptability of addressing alcohol consumption as a modifiable risk factor for breast cancer: a mixed method study within breast screening services and symptomatic breast clinics
Source: BMJ Open. 2019 Jun 17;9(6):e027371. doi: 10.1136/bmjopen-2018-027371 (PMC6609127; doi:10.1136/bmjopen-2018-027371)
Supplement: Supplementary file 1 [file bmjopen-2018-027371supp001.pdf]

Group 1
Study Number: Ethics Number:

Questionnaire

Study Title
Know Your Numbers (KYN): Adding Primary Prevention to the NHS Breast Screening Programme Phase I

Researchers: Dr Ellen Copson, Dr Julia Sinclair, Dr L. Brierley-Jones, Dr Mark McCann and Ellena Sheldon.

There are 6 sections to this questionnaire, please fill in all sections. If you have any questions, either ask the research assistant or contact us using the details on the participant information sheet.

Example: ***What colour is a clear sky?***

O *Yellow*
Ø *Blue (answer that has been selected)*
O *Purple*

Section 1. Please tick the box which most applies to you.

**Q1.**	**What is your sex?**				
	O Female		O Male		
**Q2.**	**How old are you?**				
	O 40 or under	O 41 - 50	O 51 - 60	O 61 - 70	O 71 or over
**Q3.**	**What is your ethnicity?**				
	O White British	O African	O Chinese		
	O White Irish	O Caribbean	O Bangladeshi		
	O Any other white background	O Mixed white and black Caribbean	O Mixed white and Asian		
	O Mixed white and Black African	O Any other black background	O Any other Asian background		
	O Any other ethnic group	O Not known			
**Q4.**	**What is your legal, marital or same-sex civil partnership status?**				
	O Never married or in same-sex civil partnership	O Married	O Separated but still legally married		
	O Divorced	O Widowed			

Group 1

Q5.	**What is your main occupation? (please tick one circle)**	
	O Working as an employee	O On a government sponsored training programme
	O Self-employed or freelance	O Away from work ill, on maternity leave, on holiday or temporarily laid off
	O Doing any other kind of paid work	O Retired
	O Student or in full time education	O Looking after the home or family
	O Long term sick or disabled	

Q6.	**What is your highest qualification? (tick one circle)**	
	O University degree	O CSE grades 2-5, O level D&E, GCSE grades D-G), NVQ 1-2
	O Teaching qualification or HNC/HND/BETEC higher	O CSE or GCSE ungraded
	O A or A/S levels/SCE higher or ONC/OND/BETEC/City and Guilds Advanced Final Level, NVQ 4	O Other qualifications
	O O Level or GCSE (A-C) or CSE Grade 1or City & Guilds Craft/Ord level, NVQ 3	O No qualifications

Q7.	**What are the <u>first</u> four digits of your postcode?**			

Section 2. Please tick the circle which most applies to you.

Q8.	**How is your health in general?**				
	O Very good	O Good	O Fair	O Bad	O Very bad

Q9.	**Have you ever been investigated/screened for breast cancer?** *Tick all that apply*			
	O Attended routine breast screening	O Been investigated following symptoms	O Been treated for breast cancer	O None

Q19.	**What is your….**	
	Weight? ……….	Height? ……….

Q11.	**Do you smoke?**		
	O Current smoker	O Ex-smoker	O Never smoked

UNIVERSITY OF Southampton

Group 1
Section 3.

**Q12.**	**Q14. Please write down anything that you think might increase the risk of breast cancer and then put a star by the ones you think are most important.**		
	e.g being female *		

Section 4.
We are interested to know your thoughts on the impact of adding specific cancer prevention information to the breast cancer screening process.

If a 5 minute cancer prevention information session were added to the breast screening process/ at clinic:

**Q13.**	**What do you think would be the potential advantages?** (write below)
**Q14.**	**What do you think would be the potential disadvantages?** (write below)
**Q15.**	**Would this additional 5 minute cancer prevention information session make a difference to whether you would attend screening/clinic?**
	O More likely to come to screening/ clinic<br>O Less likely to come to screening/ clinic<br>O Make no difference
**Q16.**	**What would be your preferred method for exchanging this information?**
	O it to be electronic (ie answer questions on a touch screen whilst waiting)<br>O postal<br>O trained nurse<br>O trained volunteer<br>O other – please specify………………………………………………………..

UNIVERSITY OF Southampton

Group 1
Section 5.

**Q17.**	**Do you drink alcohol?**					
	O Yes<br>O No (go to Q22.)					
**Q18.**	**Do you know how much alcohol is in a drink?**					
	O Yes<br>O No (go to Q22.)					
**Q19.**	**How would you work out or find out how much alcohol (in units) is in a drink?**					
	(Explain below)					
**Q20.**	**Quiz. Answer the questions to the nearest unit.**<br>**Answer to the best of your knowledge. Tick one correct answer.**					
**Q21.**	**A**	How many units of alcohol are in a ***medium glass of wine*** (12% ABV of 175ml)?				
		O 1	O 2	O 4	O 6	
	**B**	How many units of alcohol are in a ***pint of beer*** (4% ABV of 586ml)?				
		O 1	O 2	O 4	O 6	
	**C**	How many units of alcohol are in a ***1litre bottle of cider*** (7% of 1000ml ABV)				
		O 3	O 5	O 7	O 9	
	**D**	How many units are there in a ***standard bottle of vodka*** (40%, ABV, 700ml)?				
		O 20	O 30	O 40	O 50	

Section 6.
Focus group

We would like to invite you, and up to two female friends to a focus group to discuss more about breast cancer prevention.

Q23. Would you be interested in attending a meeting to discuss this questionnaire and the idea of adding cancer prevention to screening?

O Yes, please fill in contact details below.

O No, we do not need any contact details.

Contact details
Name:

(Please fill in at least one below)
Phone number:

Email:

Address:

Thank you very much for taking the time to complete this questionnaire.
