# Supplementary material for: The acceptability of addressing alcohol consumption as a modifiable risk factor for breast cancer: a mixed method study within breast screening services and symptomatic breast clinics
Source: BMJ Open. 2019 Jun 17;9(6):e027371. doi: 10.1136/bmjopen-2018-027371 (PMC6609127; doi:10.1136/bmjopen-2018-027371)
Supplement: Supplementary file 2 [file bmjopen-2018-027371supp002.pdf]

Appendix B – coding framework for survey responses

| | **Convincing/ probable/ limited** | **No substantial evidence** |
|---|---|---|
| **Modifiable risk factors** | Obesity/ Poor diet<br>Lack of exercise<br>Alcohol<br>Hormone medication<br>Work patterns Stress<br>smoking | Deodorants, antiperspirants<br>Underwired bras<br>Abortion,<br>Breast implants, |
| **Non- modifiable risk factors** | Family History/ genetics<br>Age<br>Height<br>Higher SE class Longer menarche<br>History of previous cancer<br>Nulliparity<br>Previous Ionising radiation<br>Female sex | Non-ionising radiation,<br>Environmental chemicals<br>Bumping or bruising the breast, |

http://www.cancerresearchuk.org/about-cancer/breast-cancer/risks-causes/risk-factors.
http://breastcancernow.org/about-breast-cancer/what-can-cause-breast-cancer
