# Supplementary material for: The acceptability of addressing alcohol consumption as a modifiable risk factor for breast cancer: a mixed method study within breast screening services and symptomatic breast clinics
Source: BMJ Open. 2019 Jun 17;9(6):e027371. doi: 10.1136/bmjopen-2018-027371 (PMC6609127; doi:10.1136/bmjopen-2018-027371)
Supplement: Supplementary pdfe 3 [file bmjopen-2018-027371supp003.pdf]

Appendix C Topic Guides

Table 1: topic guide and interview prompts

A: Topic guide for focus groups

(exact nature of these questions will be determined in part by the responses to the questionnaire - for example see question 2, where the question will be phrased based on reply to the survey)

1) **When you hear the phrase 'breast cancer risk factors' what does that mean to you? What comes to mind?**
2) **Responses to the questionnaire indicated that x, y, and z are perceived as the most significant risk factors for cancer. To what extent do you agree with these (or not)? Why (or why not)?**
3) **How do you feel about discussing/hearing about cancer and the risk factors associated with it? Is it different discussing it here compared to other settings (e,g, clinical, with family/friends/at work etc).**
4) **How do you view cancer vis-a-vis other diseases**
5) **In view of the foregoing discussion, how would you feel about introducing a 5-minute cancer prevention information session to the breast screening process/at a clinic?**

B: Interview Guide for Key Opinion Leaders and clinic staff:

(With the caveat that some of these questions will relate to/ be modified in relation to responses from the questionnaire)

1) The questionnaire shows that staff frequently/ occasionally/ never discuss breast cancer risks with patients

Why do you think that is? What makes it difficult/ easy to do this?

2) There is an initiative to add cancer prevention information to breast screening clinics- *can you tell me your thoughts on this?*

a. In your view would it deter women from attending subsequent screening. If so why?

3) What is your understanding of the relationship between alcohol and breast cancer?

4) Would you be comfortable discussing alcohol with patients? Would you be comfortable discussing LEVELS of consumption? Would you be comfortable giving advice/public health information/recommendations to reduce alcohol consumption levels?

a. If not, why not? What would make this easier? Do-able?
